# Short-Term Outcomes After Transtibial Repair of Medial Meniscus Posterior Root Tears: A Case Series

**DOI:** 10.3390/jcm14207440

**Published:** 2025-10-21

**Authors:** Dan Viorel Nistor, Samuel Piu, Diana Raluca Mihu, Romana von Mengershausen

**Affiliations:** 1 Department of Orthopedics and Traumatology, Iuliu Hațieganu University of Medicine and Pharmacy, Strada Victor Babes 8, 4000132 Cluj-Napoca, Romania; dan88nistor@elearn.umfcluj.ro (D.V.N.); piu.samuel@elearn.umfcluj.ro (S.P.); 2 Department of Orthopedics and Traumatology, County Emergency Hospital Cluj-Napoca, Strada Clinicilor 3-5, 4000347 Cluj-Napoca, Romania; diana.ralu.mihu@elearn.umfcluj.ro

**Keywords:** medial meniscus posterior root tear, meniscal root repair, transtibial pull-out technique, case series, functional outcome scores, meniscal healing, physical therapy, age factors

## Abstract

**Background/Objectives:** While arthroscopic repair is established for the treatment of medial meniscus posterior root tears (MMPRT), the relationship between physiotherapy (PT) exposure, meniscal extrusion (ME), and structural healing remains unclear. The aim of this study was to evaluate short-term functional and structural results after transtibial pull-out (TPO) repair of isolated MMPRT and to explore the influence of patient age and postoperative physiotherapy volume. **Methods:** A retrospective single-center case series with 14 adults (64% women, age 59 years, body mass index (BMI) 31.0 kg/m^2^) who underwent TPO repair (April 2022–June 2024). Mean follow-up was 18.4 months. Outcomes included range of motion (ROM), pain levels using visual analog scale (VAS), International Knee Documentation Committee (IKDC), the Western Ontario Meniscal Evaluation Tool (WOMET), the 36-Item Short Form Survey (SF-36), and MRI-based ME, cartilage grade, and root-healing status. Postoperative PT volume was assessed with a self-developed, custom questionnaire. Correlations and subgroup analyses (<60 vs. ≥60 years) were performed. **Results:** Mean postoperative ROM was 121° and IKDC 63.4. Median PT exposure was 25.9 h, and the mean duration from symptom to repair was 215 days. MRI demonstrated complete healing in 70% of cases. A positive correlation was observed between postoperative ME and ROM (*p* = 0.008), while higher PT volume was associated with greater pain scores. **Conclusions:** TPO repair appears to be a viable treatment option for selected patients with MMPRT, showing acceptable early outcomes, even in older individuals with higher BMIs or delayed repair. Meniscal healing was frequent, although extrusion progression remained common and may influence the function. The observed links between ME, ROM, and PT-related pain highlight the need for standardized rehabilitation assessment. Larger, prospective studies are warranted to validate these exploratory findings and refine postoperative management.

## 1. Introduction

Its roots are essential to the meniscus’s function of load distribution, shock absorption, lubrication, nutrition, and stabilization [1]. Lesions to the meniscus are among the most common knee injuries, with meniscal root tears accounting for 20% of all meniscal injuries [2]. A root tear is defined as the complete avulsion of the meniscus root from the tibia or a meniscal tear within 1 cm of the insertion [2]. Biomechanical studies have shown that a torn root has effects similar to a total meniscectomy and is associated with rapid progression to osteoarthritis (OA) as the articular cartilage is subjected to excessive loads [3].

The most common subtype is the medial meniscus posterior root tear (MMPRT), usually presenting as a chronic, isolated injury [2]. Magnetic resonance imaging (MRI) is the preferred imaging method. Direct signs include high signal intensity or a radial tear at the root on axial image, the Cleft sign on coronal, and the Ghost sign on sagittal sequences. Indirect signs, such as meniscal extrusion (ME) of >3 mm, subchondral bone edema, and parameniscal cysts, further support the diagnosis, although arthroscopy remains the diagnostic gold standard [2].

Arthroscopic repair techniques, and among these the transtibial pull-out (TPO) repair or suture anchor repair (SAR), are most established for the treatment of posterior meniscal root tears [2,4]. The selection of patients for surgical repair is an ongoing debate; good outcomes are reported even in patients who are not considered ideal surgical candidates [2,5,6]. Postoperative management also lacks consensus, and rehabilitation protocols vary widely [7]. Physiotherapy (PT) plays a vital role in recovery, yet its true impact and quantification are difficult to assess [8,9].

Despite numerous studies on MMPRT repair, the influence of rehabilitation intensity and the role of ME on early functional recovery remain uncertain. Understanding these relationships may help optimize postoperative care and improve patient outcomes.

Therefore, the purpose of this study was to evaluate the short-term functional and structural outcomes following TPO repair of MMPRT, focusing on subjective clinical improvement, range of motion (ROM), MRI-based healing, and ME progression, as well as the potential influence of postoperative PT exposure and patient age.

## 2. Materials and Methods

### 2.1. Patient Selection

This retrospective, single-center case series was conducted in a secondary orthopedic teaching hospital. It was approved by the Ethics Committee of the University of Medicine and Pharmacy “Iuliu Hațieganu” Cluj-Napoca (approval number: DEP279/29.22.2024).

**Figure 1:**
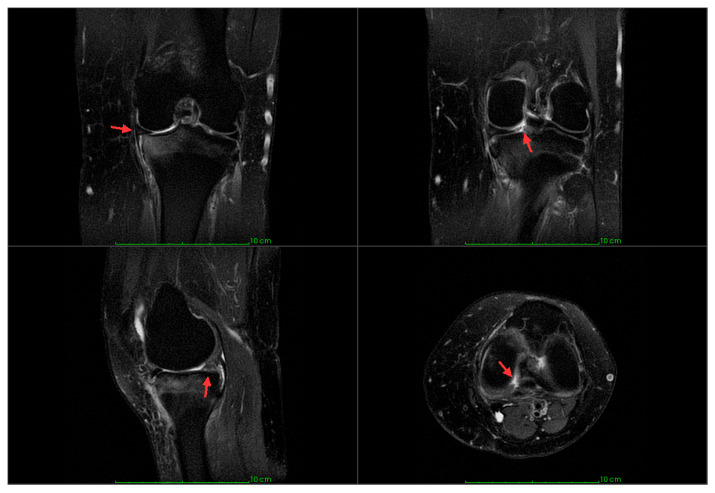
MRI findings of MMPRT. (**Upper left**) Coronal proton density-weighted fat-saturated (PD-FS) image showing meniscal extrusion (red arrow) and subchondral edema of the tibia. (**Upper right**) Coronal PD-FS image demonstrating the cleft sign (red arrow) (**Lower left**) Sagittal T2-weighted fat-saturated image with the ghost sign (red arrow). (**Lower right**) Axial PD-FS image with a radial tear at the root (red arrow).

Adult patients with MMPRT treated using the TPO technique by a single surgeon between 1 April 2022 and 30 June 2024 were identified from the hospital database. Patients undergoing combined surgical procedures, such as ligament reconstruction or lateral meniscal repair, were excluded from the study. Fourteen patients agreed to participate and were enrolled in this study. Informed consent was obtained. Information regarding age, sex, height, weight, and body mass index (BMI), symptom duration prior to surgery, and type of symptom onset was collected from the hospital records. The original diagnosis was based on MRI for 13 of those 14 patients (representative MRI findings are shown in Figure 1). The MRI was contraindicated for one patient, and a diagnostic arthroscopy was performed due to the symptoms observed during the clinical exam, which evidenced the MMPRT.

**Figure 2:**
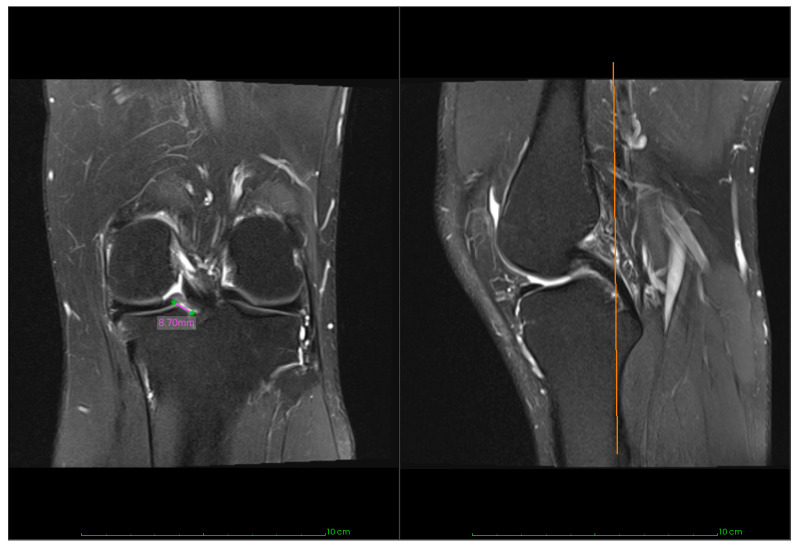
Measurement technique shown on proton density–weighted turbo spin-echo MRI with fat suppression, coronal (**left**) and sagittal (**right**) images, 3 mm slices: Straight-line distance from the anatomic medial posterior root attachment to the loose limb on the coronal slice (purple line); sagittal images are used to localize the root attachment relative to the posterior cruciate ligament tibial attachment (orange line).

Surgery was contraindicated in the cases of Outerbridge grade 4, the aspect of meniscal degeneration seen as diffuse increased intrasubstance signal intensity on T2 or meniscal contour irregularity, or a gap of ≥1 cm between the ruptured root and its insertion on the MRI image (see Figure 2). No age limit was applied, and patients with Outerbridge grade 3 cartilage changes or prolonged symptom duration were not excluded from surgical treatment.

### 2.2. Surgical Technique

The surgeries were conducted by a single orthopedic surgeon. Under spinal anesthesia, the procedures were performed with the patients positioned in dorsal decubitus with a lateral post and a foot rest installed appropriately. A tourniquet with padding was placed circumferentially on the proximal thigh with 300 mmHg pressure. Standard arthroscopic anteromedial, anterolateral portals, and an additional medial transpatellar portal were used. After routine inspection of the knee joint, the medial meniscal posterior root was assessed, and the indication confirmed. With a self-retrieving suture passing device (FIRSTPASS MINI™, Smith & Nephew, Andover, MA, USA), one or two sutures (No. 2 FiberWire^®^, Arthrex, Naples, FL, USA) were placed using the lasso method. The exact placement of the stitches depended on the quantity and quality of the tissue. The tibial tunnels were positioned anatomically with respect to the tibial attachment of the posterior cruciate ligament and the free limb of the posterior medial meniscus. Over-centralization was avoided to limit tension. The area of root insertion on the tibial plateau was thoroughly debrided using an arthroscopic shaver and ring curettes. Two tunnels were placed using an anterior cruciate ligament guide, a guiding pin, and a 4.5 mm drill. The suture ends were retrieved through the tibial tunnels and, with the knee in 90° flexion, fixated over the 10–15 mm cortical bone bridge at the proximal anterior tibial cortex level; no cortical button or supplemental anchor was used.

### 2.3. Postoperative Rehabilitation and Follow-Up

Patients were discharged on the first or second postoperative day. They were instructed to maintain non-weight-bearing (NWB) status for 4 weeks after the procedure, after which they could gradually increase the weight-bearing as tolerated. They were counseled on the importance of appropriate thromboprophylaxis and prescribed Enoxaparin following local protocol for the duration of NWB. Appropriate antalgic therapy was prescribed as needed.

Knee flexion was allowed up to a maximum of 90° for the first 4–6 weeks. No brace was used. Patients were instructed to perform isometric quadriceps contractions at home while keeping the leg in extension and actively and passively mobilize the patella in the postoperative weeks to counteract fibrosis or muscular atrophy. Physical therapy was initiated during the weight-bearing phase to restore ROM and muscular strength.

The postoperative follow-up schedule with the operating surgeon was set at six weeks, three months, six months, and one year after surgery. It was set in the same secondary care hospital where the surgery was performed. The follow-up entailed checking the wound healing progress and a knee-focused physical examination.

### 2.4. Clinical Outcomes and Imaging

At the study follow-up, patients completed several validated outcome questionnaires, including the International Knee Documentation Committee (IKDC), the Western Ontario Meniscal Evaluation Tool (WOMET), as well as the 36-Item Short Form Survey (SF-36). Passive knee ROM was measured with a goniometer as maximum flexion minus extension.

For MRI-based variables (ME, cartilage status, and healing status), complete pre- and postoperative imaging was available for only ten patients (71% of the full cohort). A postoperative MRI was obtained at a mean of 17 months (SD 7.82). Extrusion was assessed on a mid-coronal image at the meniscus midbody, as the distance between the peripheral meniscal border and the tibial plateau edge (excluding osteophytes). The cartilage was evaluated for the progression of arthrosis using the modified Outerbridge grading system [10].

To increase comparability, root healing was analyzed according to the standardized method introduced by Kim et al. [11]: On sagittal, coronal, and axial MRI, healing is categorized as complete, partial, or absent. Complete healing was defined as continuity of the root on all three planes, partial healing if any plane lacked continuity, and absent if the root was discontinuous on all three planes. All MRI scans were evaluated by an independent orthopedic surgeon experienced in musculoskeletal imaging who was blinded to the patient information.

To assess postoperative PT, a novel study-specific structured questionnaire (see Supplementary File S1) was developed, as no validated measures currently exist to quantify rehabilitation intensity after MMPRT repair. Overall PT duration, session frequency (per month and per week), and average session length are evaluated. Using the inputs from both monthly and weekly frequency, the average of the two estimates was used to approximately calculate the total PT volume (see Supplementary File S2).

### 2.5. Statistical Analysis

An a priori power analysis was performed using G*Power 3.1 to determine the minimum required sample size to detect statistically significant correlations using a biserial model (test-family: t-tests). Analysis was based on a one-tailed hypothesis, with alpha of 0.05, statistical power of 0.95, and an assumed effect size of *r* = 0.707. The study indicated a required minimum sample size of 13. Fourteen patients were enrolled.

Statistical analysis was conducted in two parts. Firstly, because preoperative subjective outcome scores were unavailable in this current study, demographic and clinical results were compared against cohort means published in similar studies using TPO repair using one-sample t-tests. More details regarding the inclusion criteria, search strategy, and statistical analysis can be found in Supplementary File S3.

Within-cohort analyses were summarized using descriptive statistics. Normality was checked with the Shapiro–Wilk test. Correlations were analyzed using Pearson or Spearman tests, and group differences were assessed with t-tests or the Mann–Whitney U test for continuous data. At the same time, categorical variables were compared with Fisher’s exact or chi-square test. Linear regression explored significant associations. For MRI-based variables, ten patients were available and analyzed separately.

All statistical analyses were performed using IBM SPSS Statistics, version 26, and Python v3.13. The significance level was set at *p* < 0.05.

## 3. Results

At a mean follow-up of 18 months, the mean IKDC score was 63.4; the mean ROM was 121°; and 70% of repairs showed complete healing on MRI. 79% of patients attended PT, with an average of 25.9 h of total PT volume. The cohort consisted of nine female and five male patients for most of the variables. The primary demographic and clinical data are presented in Table 1.

### 3.1. Comparative Results

An overview of the studies included in the comparative analysis, their mean variables, and the *p*-values resulting from the one-sample t-test are presented in Table S3 (see Supplementary File S3). As outlined in the detailed description of the comparative statistical analysis in Supplementary File S3, forest plots were limited to studies that reported both mean and standard deviation.

**Figure 3:**
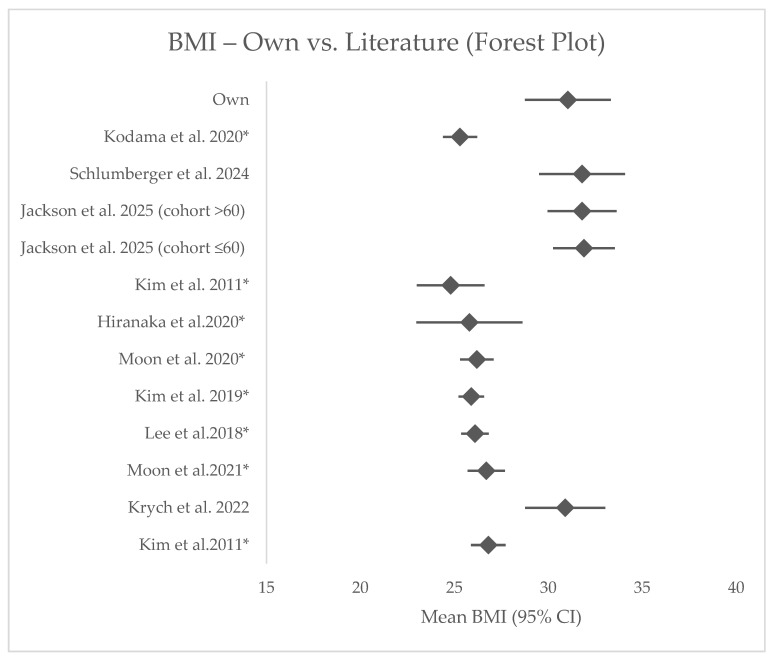
Forest plot illustrating the effect sizes and 95% confidence intervals for the BMI variable across included cohorts (BMI—body mass index). Statistically significant differences (*p* < 0.05) are marked with * [6,11,12,13,14,15,16,17,18,19,20].

**Figure 4:**
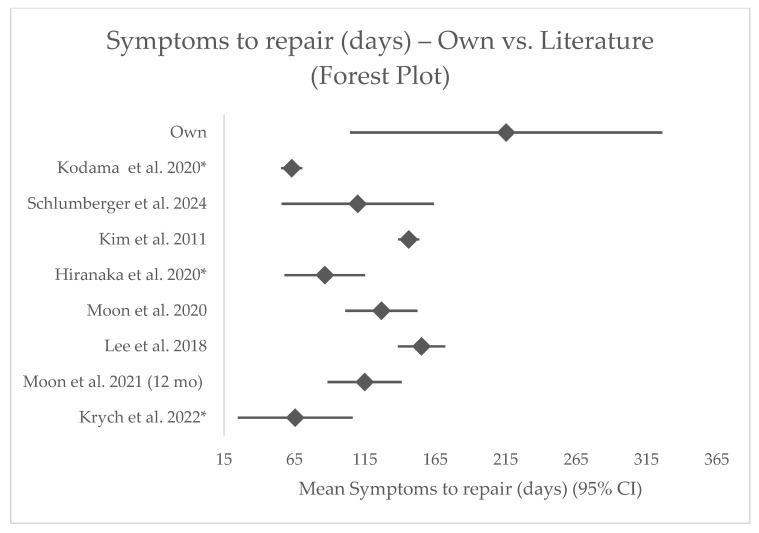
Forest plot illustrating the effect sizes and 95% confidence intervals for the symptom-to-repair variable across included cohorts. Statistically significant differences (*p* < 0.05) are marked with * [6,12,13,15,16,17,18,20].

Across 14 comparator cohorts, this series had a similar age but higher BMI and longer symptom-to-repair intervals (Table S3, Figure 3 and Figure 4). The postoperative IKDC score fell within the mid-range of published values. In the reduced MRI subset of 10 patients, ME values were broadly comparable, with some patients reporting lower extrusion (Table S3).

### 3.2. Within-Cohort Results

Table 2 summarizes the results of the subjective outcome scores applied; 50% of patients were ≥60 years. No statistically significant differences were found between patients aged < 60 years and ≥60 years for any clinical outcomes and any of the evaluated MRI-related outcomes.

**Figure 5:**
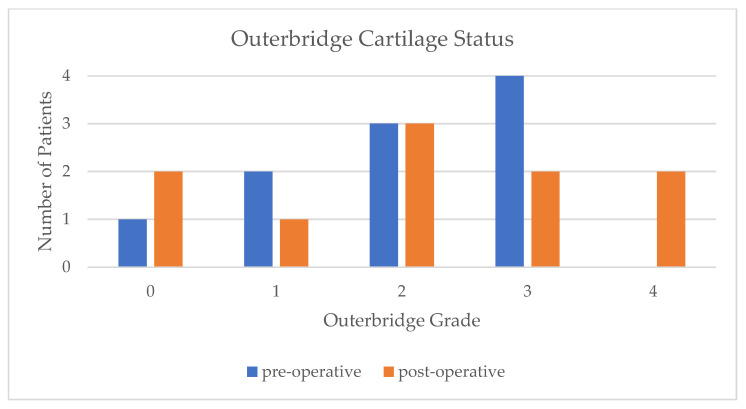
Bar chart comparing cartilage status (Outerbridge classification) preoperatively and postoperatively.

On the MRIs, pre- and postoperative ME (see Table 1), cartilage status (Figure 5), and the meniscal healing status on the postoperative images were analyzed. The results showed that 70% of the cases exhibited complete healing, 10% partial healing, and 20% absent healing.

When analyzing the correlation between variables, significant and clinically relevant associations were identified between postoperative ME and ROM (r = 0.781, *p* = 0.008) and between total PT hours and pain levels at follow-up (rho = 0.723, *p* = 0.018). Both relationships were further quantified with linear regression analysis, which indicated that postoperative ME was a significant predictor of ROM at follow-up (B = 7.58, SE = 2.15, Beta = 0.78, *p* = 0.008). The regression model was statistically significant (F = 12.49, *p* = 0.008) and explained 61% of the variance in ROM (R^2^ = 0.61). Average total PT hours was identified as predicting pain scores at follow-up (B = 0.05, SE = 0.02, Beta = 0.68, *p* = 0.031). The model explained 46% of the variance in pain scores (R^2^ = 0.46, F = 6.85, *p* = 0.031). No additional statistically significant or clinically relevant associations were found.

## 4. Discussion

This study found that TPO repair of MMPRT achieved acceptable short-term functional outcomes, with 70% of patients showing complete healing on MRI. ME tended to progress and correlated positively with ROM, while greater PT exposure was associated with higher reported pain.

With a mean age of 59 years and a BMI of 31 kg/m^2^, this patient is representative of the demographic suffering from MMPRT [2]. Despite higher average BMI values and longer symptom-to-repair period, the postoperative IKDC was in the mid-range of many published studies [12,13,14,15]. No age-related differences in outcomes were observed, consistent with findings that older age alone should not be considered a contraindication for MMPRT repair [21,22,23].

Arthroscopic repair shows better outcomes compared to conservative treatment or meniscectomy and is considered the most cost-effective strategy to delay OA progression [24,25,26,27,28]. However, even with subjectively successful repair, ME often persists or progresses, as observed in this cohort, and has been associated with an increased risk of degenerative changes [5,16,29,30]. Long-term studies have identified pre-operative varus alignment and postoperative ME as predictors of clinical failure [30], emphasizing the importance of biomechanical restoration. Complete healing in this series occurred in 70% of cases, which falls within the mid-range of values reported by Feucht et al. [31], highlighting the variability of biological healing even with consistent, acceptable clinical outcomes. Several surgical factors implicated in these unfavorable findings have been proposed and tested, including the placement of transtibial tunnels, suture factors, and the timing of the repair [17,32,33,34,35].

Although early repair has been linked to reduced extrusion progression [16], no such association was observed in this cohort, possibly due to the sample size and inclusion of chronic presentations. Interestingly, a positive correlation was found between extrusion and ROM, which should be interpreted with caution. The small MRI sample size increases the likelihood that this finding reflects spurious or sample-related correlation rather than true physiological association. This finding is noteworthy, as knee ROM is not routinely measured as part of the clinical outcome of MMPRT repair, nor is it included in the statistical testing, representing an underexplored clinical parameter.

Postoperative rehabilitation varies across the literature, with both conservative and accelerated protocols demonstrating comparable outcomes and no established gold standard [7,36]. PT plays an essential role in postoperative recovery, but is difficult to measure due to variability in adherence, therapy quality, and external factors [8]. While reported compliance averages at 60% [8,37], 79% of patients in this cohort attended PT, consistent with evidence that postoperative patients are more likely to engage in rehabilitation [37]. In this study, PT exposure was quantified using a study-specific questionnaire, as no validated tool was available. A significant correlation was observed between greater PT volume and higher pain scores at follow-up (*p* = 0.031), which may reflect reverse causality rather than a direct treatment effect, as patients with more symptoms are more likely to receive more therapy. Yet, these exploratory findings underscore the need for future studies to include validated measures of PT adherence and assess whether standardized protocols can improve pain and function after MMPRT repair.

This study has several limitations, including a retrospective design, small sample size, single-center setting, lack of controls, missing preoperative PROMs, and single-examiners for clinical and imagistic assessments, all of which reduce generalizability and introduce bias.

One important limitation is the assessment of PT exposure using a custom, patient-reported form with uncertain reliability and accuracy, as non-validated tools introduce potential recall bias and limit reproducibility. The total PT volume was calculated using approximations and the average of the reported frequencies per month and per week, a method implemented to increase estimation robustness, but it needs to be refined and formally validated. Additionally, pure quantification of PT hours does not contribute to assessing session quality and true adherence. Given these limitations, the PT data should be considered pilot, exploratory, and hypothesis-generating, highlighting the need for standardized and validated tools for PT exposure assessment. Prospective development and validation require a dedicated line of research.

Despite these weaknesses, possible new insights are provided into the association between ROM and ME postoperatively, as well as the relation between the total volume of PT and pain levels. By comparing our data to the literature, we confirmed that the generally reported improvement in subjective outcome measures is consistent, as well as the objective variables of increased ME, variable root healing status, and OA progression.

These findings highlight that while patients appear to benefit from meniscal root repair, there is room for improvement.

No randomized controlled trials (RCTs) have been conducted to compare MMPRT repair with alternative treatment options. Root tear treatment is based on systematic reviews and meta-analyses of level 3 and 4 research [24,26,27]. One RCT (NCT05985772) comparing TPO repair with structured conservative treatment is currently underway [38].

Several emerging considerations and techniques are likely to influence the future of MMPRT management. Auxiliary procedures in addition to classic root repair have been introduced [39]. Some advocate for centralization sutures, some authors propose high tibial osteotomy [29,30,39]. Another promising area is the use of biologic augmentation techniques to enhance meniscal healing. Approaches in this area range from preclinical research to techniques already implemented in clinical practice, such as platelet-rich plasma or gel (PRP or PRG), and many more [40]. Recent RCTs by Kaminski et al. [41,42] demonstrated improved healing and functional outcomes when PRP and bone marrow venting were used in conjunction with meniscal tear repair. Further research is required to assess the potential role of auxiliary procedures or orthobiologics in the future of meniscal root repair.

## 5. Conclusions

In this exploratory, single-surgeon series, transtibial pull-out repair of MMPRT produced acceptable short-term clinical outcomes despite high BMI and prolonged symptom-to-repair intervals. Meniscal healing was frequent, whereas ME tended to progress. No age-related differences were detected in clinical or imaging outcomes. Observed associations between ME and ROM or PT volume and pain should be interpreted cautiously. Larger, prospective studies are warranted to confirm these pilot findings and standardize rehabilitation and physiotherapy assessment.

## Figures and Tables

**Table 1:** Basic features of the studied group.

	*N*	Mean ± SD
Follow-up time (months)	14	18 ± 8
Age (years)	14	59 ± 13
BMI (kg/m^2^)	14	31 ± 4
Symptom-to-repair (days)	14	215 ± 212
ROM (degrees)	14	121 ± 16
ME preoperative (mm)	10	3.32 ± 1.77
ME postoperative (mm)	10	3.94 ± 1.72

BMI—body mass index; ROM—range of motion; ME—meniscal extrusion.

**Table 2:** Subjective outcomes scores.

Outcome	Mean ± SD
Pain (VAS)	2.79 ± 3.22
IKDC	63.4 ± 18.8
WOMET total aggregate	62.6 ± 31.1
SF-36 points	80.7 ± 19.4

VAS—visual analog scale; IKDC—International Knee Documentation Committee; WOMET—Western Ontario Meniscal Evaluation Tool; SF-36—36-Item Short Form Survey.

## Data Availability

The data generated and analyzed in the present study are available upon reasonable request from the corresponding author due to concerns regarding privacy and ethical reasons. Aggregate data supporting the main findings are included within the article.

## Supplementary Materials

The following supporting information can be downloaded at: https://www.mdpi.com/article/10.3390/jcm14207440/s1. File S1: Postoperative physiotherapy volume questionnaire, File S2: Calculation of total PT volume, File S3: Comparative statistical analysis and list of comparative studies. Refs. [43,44] are cited in File S3.

## Abbreviations

The following abbreviations are used in this manuscript:

BMI	Body mass index
IKDC	International Knee Documentation Committee
ME	Meniscal extrusion
MMPRT	Medial meniscus posterior root tear
NWB	Non-weight bearing
OA	Osteoarthritis
PD-FS	Proton density-weighted fat-saturated
PROM	Patient-reported outcome measure
PRP/PRG	Platelet-rich plasma/gel
PT	Physiotherapy
RCT	Randomized controlled trial
ROM	Range of motion
SAR	Suture anchor repair
SF-36	36-Item Short Form Survey
TPO	Transtibial pull-out technique
VAS	Visual analog scale
WOMET	Western Ontario Meniscal Evaluation Tool
