# Supplementary material for: Short-Term Outcomes After Transtibial Repair of Medial Meniscus Posterior Root Tears: A Case Series
Source: J Clin Med. 2025 Oct 21;14(20):7440. doi: 10.3390/jcm14207440 (PMC12565521; doi:10.3390/jcm14207440)
Supplement: Supplementary file 1 [file jcm-14-07440-s001.zip › Supplementary_File_S1.pdf]

(Table S1)

English translation:

Postoperative Physiotherapy (PT) Volume Questionnaire

Name:___________________________

Date:_________________

1. Where did you perform PT?
   - Inpatient
   - Outpatient
   - At home
   - Combination

2. What type of PT did you perform? (You may choose more than one)
   - Cryotherapy
   - Electrotherapy
   - Therapeutic massage
   - Ultrasound therapy
   - Kinesiotherapy
   - Other: ___________________

3. How many months after surgery did you continue PT?

<1	1-2	3-4	5-6	7-8	9-10	11-12	>12

4. How would you describe the regularity of PT after the intervention?
   - Very regular
   - Regular
   - Fairly regular
   - Variable
   - Very variable

5. During the period defined in question 3 — How many times per month? *(Please mark the number and description that best apply. If activity intensity changed, mark two numbers: the highest and the lowest.)*

1	2	3	4	5	6	7	8	>8

   - Several times per week
   - Every week
   - Every second week
   - Once per month

6. During the period defined in question 5 — How many times per week? *(If activity intensity changed, mark two numbers: the highest and the lowest.)*

1	2	3	4	5	6	7	>7

7. What was the average duration of a PT session?

<30 min	30 min	1 oră	1,5 ore	2 ore	>2 ore

8. Why did you stop?
   - o Financial reasons
   - o Time
   - o Ineffective
   - o Recovery
   - o Other: ____________________________

Original Romanian wording:

Quantificarea Fiziokinetoterapie (FKT)

Evaluarea a Duratei și Cantității

Nume:___________________________

Data:_________________

1. Unde ați efectuat FKT?
    - o Spitalizare
    - o Ambulatoriu
    - o Acasă
    - o Combinație

2. Ce tip de FKT ați efectuat? (Puteți alege mai multe)
    - o Crioterapie
    - o Electroterapie
    - o Masaj therapeutic
    - o Terapia cu ultrasunete
    - o Kinetoterapie
    - o Altele:_________________

3. Câte luni după operație ați continuat FKT?

<1	1-2	3-4	5-6	7-8	9-10	11-12	>12

4. Cum ați descrie evoluția FKT după intervenție?
    - o Foarte regulată
    - o Regulat
    - o Destul de regulată
    - o Variabilă
    - o Foarte variabilă

5. În perioada definită la punctul 3) - De câte ori pe lună? (Vă rog să marcați numărul și descrierea celei mai potrivite. Dacă există schimbări în activitatea de intensitate mare/ scăzută, marcați două numere: cea mai mare și cea mai mica.)

1	2	3	4	5	6	7	8	>8

- o De mai multe ori pe săptămână
- o Fiecare săptămână
- o Fiecare a doua săptămână
- o O dată pe lună

6. În perioada definită la punctul 5) - De câte ori pe săptămână? Dacă există schimbări în activitatea de intensitate mare/ scăzută, marcați două numere: cea mai mare și cea mai mică?

1	2	3	4	5	6	7	>7

7. Care a fost durata medie a sesiunii de FKT?

<30 min	30 min	1 oră	1,5 ore	2 ore	>2 ore

8. De ce ați încetat:
   - Motive financiare
   - Timp
   - Ineficient
   - Recuperare
   - Altele: ______________________________
