# Supplementary material for: Short-Term Outcomes After Transtibial Repair of Medial Meniscus Posterior Root Tears: A Case Series
Source: J Clin Med. 2025 Oct 21;14(20):7440. doi: 10.3390/jcm14207440 (PMC12565521; doi:10.3390/jcm14207440)
Supplement: Supplementary file 1 [file jcm-14-07440-s001.zip › Supplementary_File_S2.pdf]

Table S2. Calculation of Total PT Volume.

Method	Formula
Monthly frequency	*PT Hours* = Duration (months) × Sessions per Month × Hours per Session
Weekly frequency	*PT Hours* = Duration (months) × 4.3 (weeks/month) × Sessions per Week × Hours per Session
**Total PT volume**	*Total PT Hours* = (Monthly frequency + weekly frequency)/2

PT = Physiotherapy.
