# Supplementary material for: Short-Term Outcomes After Transtibial Repair of Medial Meniscus Posterior Root Tears: A Case Series
Source: J Clin Med. 2025 Oct 21;14(20):7440. doi: 10.3390/jcm14207440 (PMC12565521; doi:10.3390/jcm14207440)
Supplement: Supplementary file 1 [file jcm-14-07440-s001.zip › Supplementary_File_S3.pdf]

Supplementary File S3 - Description of the Comparative Statistical Analysis and List of Comparative Studies

To contextualize the outcomes of the patient cohort in light of lacking preoperative subjective data, a targeted literature search was conducted to identify suitable studies for comparison. Case series, case-control, or other cohort studies investigating patients undergoing the same surgical procedure were included. Studies were eligible if they reported postoperative IKDC, age, and involved a short-term follow-up. Only the cohorts receiving arthroscopic TPO repair were considered relevant for direct comparison.

Since published studies provide only summary statistics comprised of mean ± standard deviation and sample size, a one-sample t-test approach was used for comparative analysis. Demographic (age, BMI) and clinical variables (symptom duration, ROM, IKDC, ME) were compared with those reported in published studies for each eligible study. All studies with reported mean values were included in the one-sample t-test comparison. In contrast, only studies reporting both mean and standard deviation for the respective variable were represented in forest plots, as they are necessary to visualize confidence intervals. Studies that lacked complete summary data for a specific variable were excluded from that analysis but could be included for other variables if sufficient data were available. This allowed the analysis whether this cohort differed significantly from the outcomes reported in published studies, without raw data from the literature groups.

Table S3. List of Comparative Studies.

No.	Study	N	Age (y)	p-value	BMI (kg/m^2)	p-value	IKDC	p-value
**1**	Kodama et al. [18]	47	63.7	0.1749	25.3	0.0003*	70.5	0.1782
**2**	Schlumberger et al. [13]	38	49.6	0.0188*	31.8	0.5260	63.0	0.9432
**3**	Jackson et al. [14]	20	65.7	0.0646	31.8	0.5260	65.4	0.6913
**4**	Jackson et al. [14]	40	49.3	0.0159*	31.9	0.4741	66.7	0.5175
**5**	Kim et al. [12]	23	52.8	0.1040	24.8	0.0001*	94.4	<0.0001*
**6**	Rocha de Faria et al. [43]	22	53.1	0.1216	/	/	69.8	0.2217
**7**	Hiranaka et al. [17]	25	62.5	0.2978	25.8	0.0006*	70.5	0.1782
**8**	Moon et al. [16]	63	54.9	0.2770	26.2	0.0012*	55.7	0.1503
**9**	Kim et al. [19]	21	55.9	0.4147	25.9	0.0007*	75.2	0.0345*
**10**	Lee et al. [15]	56	53.3	0.1332	26.1	0.0010*	64.9	0.7642
**11**	Bernard et al. [44]	15	46.1	0.0027*	32.0	0.4264	72.3	0.0981
**12**	Moon et al. [20]	51	55.5	0.3548	26.7	0.0026*	56.6	0.2003
**13**	Krych et al. [6]	45	42.3	0.0003*	30.9	0.9080	79.5	0.0067*
**14**	Kim et al. [11]	30	55.2	0.3141	26.81	0.0031*	74.1	0.0518

BMI – Body mass index; IKDC – International Knee Documentation Committee.
Statistically significant differences ($p < 0.05$) marked with *.

Table S3. List of Comparative Studies continued.

No.	Symptom-to-repair (days)	p-value	ME preoperative (mm)	p-value	ME postoperative (mm)	p-value
**1**	63.0	0.0185*	/	/	/	/
**2**	109.9	0.0854	/	/	/	/
**3**	/	/	/	/	/	/
**4**	/	/	/	/	/	/
**5**	146.1	0.2435	4.10	0.154	2.20	0.011*
**6**	/	/	/	/	/	/
**7**	86.5	0.0405*	/	/	/	/
**8**	126.7	0.1417	3.40	0.758	4.40	0.420

**9**	/	/	/	/	/	/
**10**	114.8	0.3080	/	/	/	/
**11**	/	/	/	/	/	/
**12**	114.8	0.0993	3.50	0.633	4.50	0.330
**13**	65.5	0.0202*	1.90	0.045*	2.60	0.036*
**14**	/	/	3.13	0.877	2.94	0.099

ME – meniscal extrusion. Statistically significant differences ($p < 0.05$) marked with *.
